# Non-canonical DNA/RNA structures associated with the pathogenesis of Fragile X-associated tremor/ataxia syndrome and Fragile X syndrome

**DOI:** 10.3389/fgene.2022.866021

**Published:** 2022-08-30

**Authors:** Aadil Yousuf, Nadeem Ahmed, Abrar Qurashi

**Affiliations:** Department of Biotechnology, University of Kashmir, Srinagar, Jammu and Kashmir, India

**Keywords:** fragile X-associated tremor/ataxia syndrome (FXTAS), fragile X syndrome (FXS), FMR1, R-loop, hairpin

## Abstract

Fragile X-associated tremor/ataxia syndrome (FXTAS) and fragile X syndrome (FXS) are primary examples of fragile X-related disorders (FXDs) caused by abnormal expansion of CGG repeats above a certain threshold in the 5′-untranslated region of the fragile X mental retardation (FMR1) gene. Both diseases have distinct clinical manifestations and molecular pathogenesis. FXTAS is a late-adult-onset neurodegenerative disorder caused by a premutation (PM) allele (CGG expansion of 55–200 repeats), resulting in FMR1 gene hyperexpression. On the other hand, FXS is a neurodevelopmental disorder that results from a full mutation (FM) allele (CGG expansions of ≥200 repeats) leading to heterochromatization and transcriptional silencing of the FMR1 gene. The main challenge is to determine how CGG repeat expansion affects the fundamentally distinct nature of FMR1 expression in FM and PM ranges. Abnormal CGG repeat expansions form a variety of non-canonical DNA and RNA structures that can disrupt various cellular processes and cause distinct effects in PM and FM alleles. Here, we review these structures and how they are related to underlying mutations and disease pathology in FXS and FXTAS. Finally, as new CGG expansions within the genome have been identified, it will be interesting to determine their implications in disease pathology and treatment.

## Introduction

**FIGURE 1:**
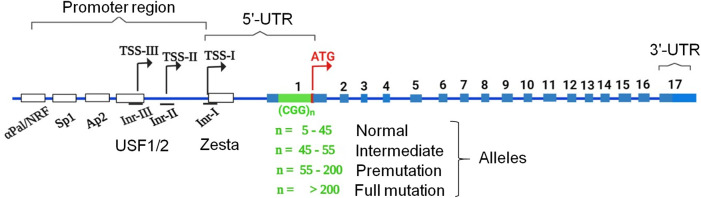
Representation of the canonical structure of the FMR1 gene and its alleles (normal, intermediate, PM, FM) as a result of CGG repeat expansion in the 5′-UTR. Exons 1 to 17 that can be spliced in different ways, as well as sites for binding transcription factors and transcription start sites (TSS-I, TSS-II, and TSS-III).

CGG repeats are a type of microsatellite or short tandem repeat (STR) found in the human genome, with the majority located in the 5′-untranslated regions (5′-UTRs), suggesting that they may play a role in transcriptional regulation or translation initiation (Bagshaw, 2017). Abnormal expansion of CGG repeat tracts above a certain threshold confers instability and chromosome fragility, resulting in various clinical manifestations. CGG expansion in the FRAXA (folate-sensitive fragile site, X chromosome, A) region has distinct effects on the fragile X mental retardation1 (FMR1) gene located on the X chromosome (Xq27.3) (Verkerk et al., 1991). The structure of FMR1 is shown in Figure 1. It contains 56 CpG sites spread across 1 kb of its promoter and a naturally occurring CGG triplet-repeat region in its first exon (Oberlé et al., 1991; Naumann et al., 2014). However, in the general population, there are some polymorphisms in the CGG repeat region in terms of the length and content of AGG repeats, which are often interspersed with a periodicity of the 9th to 11th repeats. AGG interruptions significantly increase the stability of CGG repeats (Nolin et al., 2013; Yrigollen et al., 2014). Carriers of FMR1 alleles that are either normal (<55 repeats) or have 55–200 repeats (premutation (PM) alleles) have much lower rates of chromosomal fragility. Longer CGG repeats are extremely unstable during intergenerational transmission (Nolin et al., 2019) and in somatic cells, resulting in CGG repeat expansion (Lokanga et al., 2013). Therefore, chromosome fragility is prominent in carriers of the FMR1 allele with massive CGG repeat expansions of >200 repeats (full mutation (FM) alleles) (Mila et al., 2018). The FM allele is usually accompanied by heterochromatization, transcriptional silencing, and subsequent loss of FMR1 protein (FMRP) expression, resulting in fragile X syndrome (FXS; OMIM #300624) (Mila et al., 2018). FXS is the most common form of inherited intellectual disability (ID) and is the leading genetic cause of autism (Hagerman et al., 2010). However, PM alleles are associated with transcriptional increases in FMR1, which could be related to euchromatization of the FMR1 locus and an upstream shift in the transcription start from the transcription start site (TSS-I) of FMR1 (Tassone et al., 2000; Hagerman, 2012; Schneider et al., 2020). Such hyperexpression of the PM allele paradoxically leads to a relatively normal or gradual reduction in FMRP with increasing repeat length (Hagerman and Hagerman, 2004). Hyperexpression of PM alleles is associated with specific disorders, including fragile X premature ovarian insufficiency (FXPOI; OMIM #311360), a condition associated with menopause in women aged <40 years (Sullivan et al., 2011; Sherman et al., 2014), and fragile X-associated tremor/ataxia syndrome, a neurodegenerative disorder (FXTAS; OMIM #300623) that affects PM carriers, mostly men over the age of 50 years, with clinical manifestations such as action tremors, gait ataxia, Parkinsonism, and cognitive decline (Hagerman and Hagerman, 2016). In model systems, hyperexpression of riboCGG repeats in the PM range leads to defects in cell development and cell toxicity (Hagerman, 2012; Hagerman et al., 2018; Bhat et al., 2021). Unlike FM alleles, PM alleles alter RNA-processing mechanisms, which may be related to unusual secondary structures formed by DNA strands and RNA containing CGG and antisense CCG repeats (Zhao and Usdin, 2021). Such unusual secondary structures can potentially impede translation within the PM range through an obscure mechanism (Zhao and Usdin, 2021). In addition, such secondary structures can sequester specific proteins from their normal biological functions and/or undergo repeat-associated non-AUG (RAN) translation from both sense and antisense strands into toxic homopolymeric peptides (Todd et al., 2013). Homopolymeric peptides, such as polyGlycine (FMRpolyG), have been identified in neuronal inclusions of FXTAS patients (Buijsen et al., 2014). Additionally, FMRpolyG overexpression is toxic to cells in various FXTAS model systems (Kearse et al., 2016; Sellier et al., 2017). This review focuses on how secondary structures are related to the PM and FM alleles and their associated diseases. Recent years has seen a flurry of papers reporting novel CGG repeat expansions within the genome and some have been cloned and associated with neurodevelopmental or neurodegenerative diseases (Deng et al., 2019; Ishiura et al., 2019; Okubo et al., 2019; Sone et al., 2019; Tian et al., 2019; Jiao et al., 2020; Ma et al., 2020; Sun et al., 2020; Annear et al., 2021). Some share common genetic and clinical features, allowing for a better understanding of disease mechanisms and development of therapeutic strategies.

### Structural polymorphism of CGG/CCG repeats in the FMR1 gene

**FIGURE 2:**
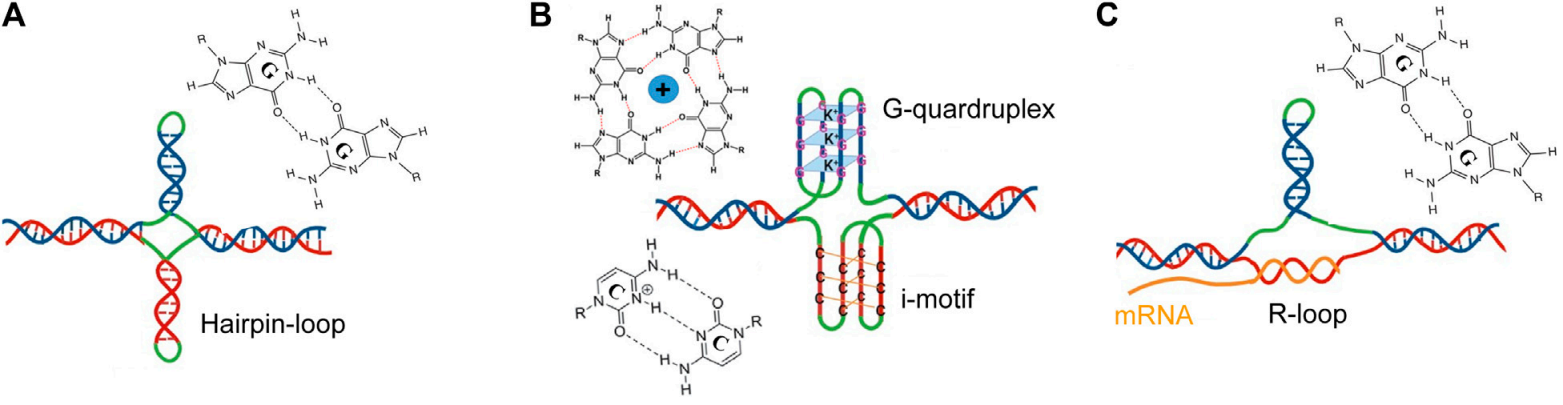
Representation of non-canonical secondary structures formed by CGG (blue) or CCG (red) repeat expansions on the respective strands of FMR1. **(A)** Hairpin created on sense (blue) and antisense (red) strands, **(B)** a G-quadruplex formed on a sense (blue) strand or an i-motif structure formed on the antisense strand (red), and **(C)** an R-loop formed by the annealing of nascent RNA and non-template strand (red). The unpaired loops are shown in green.

As shown in Figure 2, individual strands of expanded CGG repeats form a variety of stable non-canonical DNA and RNA structures during processes involving transient DNA unwinding such as replication, repair, transcription, and/or recombination. There is conflicting evidence regarding the secondary structural preference of DNA and RNA strands. CGG stem-loop/hairpins are relatively stable and easily formed *in vitro* and *in vivo* using Watson-Crick G:C and Hoogsteen G:G base pairs (Figure 2A) (Chen et al., 1995; Mitas et al., 1995; Nadel et al., 1995; Usdin and Woodford, 1995; Yu et al., 1997; Handa et al., 2003; Sobczak et al., 2003; Zumwalt et al., 2007; Ciesiolka et al., 2017; Ajjugal et al., 2021; Poggi and Richard, 2021). However, in the presence of physiological K+ concentrations, stable G-quadruplex (G4) and intercalated-motif (i-motif) structures are formed from CGG and CCG repeat strands, respectively (Figure 2B) (Kettani et al., 1995; Fojtík and Vorlícková, 2001; Weisman-Shomer et al., 2002; Weisman-Shomer et al., 2003; Khateb et al., 2007; Renčiuk et al., 2011; Krzyzosiak et al., 2012; Loomis et al., 2014; Malgowska et al., 2014; Yang and Rodgers, 2014; Chen et al., 2018; Asamitsu et al., 2021). The formation of hairpins or tetrahelical structures (dimerization of hairpins) is altered by AGG interruption and cell type (Jarem et al., 2010). The strands of CCG repeats are also unpaired and form stable pathological R-loops, which are RNA:DNA hybrid duplexes that are formed in the transcribed region during transcription (Figure 2C) (Abu Diab et al., 2018; Crossley et al., 2019). A hairpin in the non-template strand could reduce duplex reannealing behind the advancing transcription complex, and thus aid in R-loop formation. The persistence of the R loop, on the other hand, may favor the development of the hairpin on the non-template strand. R-loop structures composed of a G-rich RNA template and C-rich DNA template are thermodynamically advantageous and stable compared to DNA duplexes (Roberts and Crothers, 1992; Belotserkovskii et al., 2013; Takahashi and Sugimoto, 2020). Interestingly, unlike hairpins, the formation of the R-loop is not affected by AGG interruption within the CGG repeat tract (Reddy et al., 2011) suggesting that they are formed in most repeat expansion disorders (REDs) that become heterochromatinized.

### CGG/CCG repeat associated secondary structures play a role in the expansion of CGG repeats in the FMR1 gene

**FIGURE 3:**
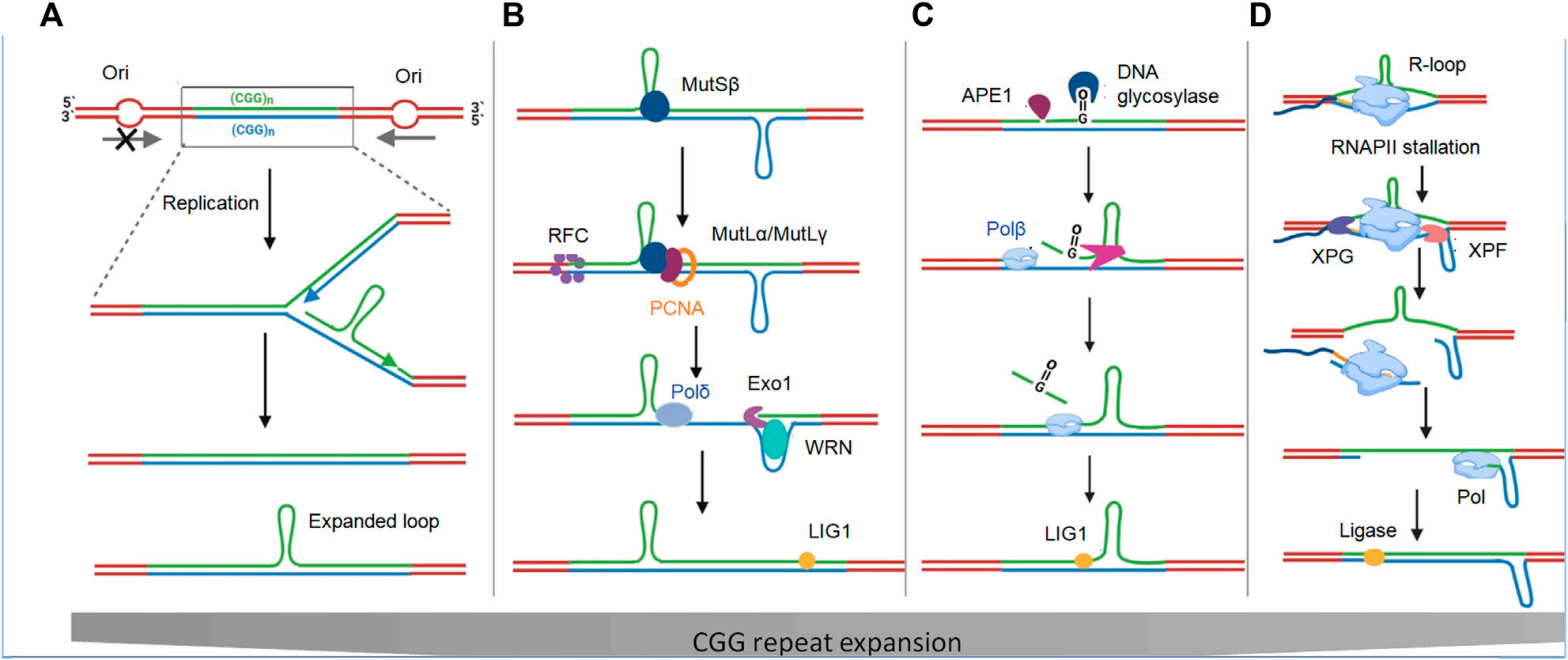
Repeat instability models. **(A)** Model of repeat instability based on the Ori-switch. The absence of replication ORI upstream of the CGG repeat track causes formation of hairpin-like secondary structures on the lagging strand, leading to polymerase slip and resulting in repeat expansion in the new daughter strand. **(B)** Model of repeat instability based on mismatch repair (MMR). Repeat instability occurs by causing a nick at the base of loopouts that are bound by mismatch repair factors MutSβ or MutLγ, and are processed *via* a DSB to generate expansions. MutLγ endonuclease activity can be directed by a nick to cleave the opposite strand in a concerted manner to create a DSB. Out-of-register annealing could result in the activation of *via* the non-homologous end joining (NHEJ) or microhomology-mediated end joining (MMEJ) pathway. **(C)** Base excision repair (BER) model of repeat instability. DNA glycosylase recognizes the oxidized base and APEI creates a nick, leaving a single-stranded break. Strand slippage results in the formation of repeat-associated hairpins either on the lesion or on the opposite non-lesion strand, leading to a multinucleotide gap. **(D)** Nucleotide excision repair (NER) model of repeat instability. RNA polymerase stalls because of R-loop formation and/or the formation of secondary structures on the non-template strand. Stalled transcription recruits transcription arrest factors, including CSB and XPG, which nicks the repeated region at two different sites, and thus removes this fragment. DNA pol then refills this gap *via* transcription-coupled-NER (TC-NER). Repetitive regions in DNA (green) and RNA (yellow) are shown.

FMR1 is flanked by two origins of replication (ORIs): One 45 kb upstream and one 45 kb downstream (Gerhardt et al., 2014). Inactivation of upstream ORIs in FM human embryonic stem cells (hESCs) and PM cells most likely occurs during germ cell generation and the early stages of embryogenesis when rapid cell division and more ORIs are simultaneously required to complete genome replication. During replication, hairpin and tetrahelical structures have been observed to pause DNA polymerases in both *in vitro* and *in vivo* studies (Viguera et al., 2001; Murat et al., 2020), resulting in the probability of replication irregularities and repeat instability. When such structures are formed on the Okazaki fragments of the lagging strand, the polymerase slips, resulting in the expansion of repeats in the daughter strand (Figure 3A). In contrast, a hairpin on the template of the leading strand causes the polymerase to skip the loop, resulting in contraction of the repeat in the daughter strand (Kim and Mirkin, 2013). Replication difficulties may also explain why offspring from male PM carriers do not inherit expanded or FM alleles. This is because, unlike post-mitotic oocytes, sperm cells undergo multiple rounds of replication before fertilization, which could provide selective pressure for expansion in male PM carriers compared to female PM carriers.

Repeat expansion can also occur during the repair of secondary structures through redundant repair events that are not protective but are harmful either by leading to repeat expansion or contraction (Salinas-Rios et al., 2011; Pluciennik et al., 2013). Genome-wide association studies (GWAS) in patient cohorts with various repeat expansion disorders (REDs) have implicated a variety of mismatch repair (MMR) proteins such as mutS homolog 3 (MSH3), mutL homolog 1 (MLH1), and mutL homolog 3 (MLH3) as important modifiers of repeat expansion and disease severity. These proteins are required for repeat expansion in FXDs and in several RED mouse models (Schmidt and Pearson, 2016; Kadyrova et al., 2020). For example, in an FX PM mouse model, overexpression of MSH2 increased the frequency of both intergenerational CGG repeat expansion and somatic expansion, whereas ablation of MSH2 reduced both repeat number and expansion frequency in a dose-dependent manner (Lokanga et al., 2013; Lokanga et al., 2014). Similarly, in mESCs derived from FX PM mice, the point mutation D1185N in the endonuclease domain of MLH3 precludes repeat expansion, suggesting its importance in this process (Hayward et al., 2020). In addition to MutSβ (an MSH2 and MSH3 heterodimer) and MutSγ (an MSH2 and MSH6 heterodimer) (López Castel et al., 2010; Zhao et al., 2015; Zhao et al., 2016), three other mammalian protein complexes, MutSα, MutLα, and MutLβ, play important roles in expansion (Figure 3B) (López Castel et al., 2010; Miller et al., 2020; Zhao and Usdin, 2021). Although these studies have suggested that the MMR pathway plays a role in repeat expansion, the mechanism by which MMR substrates are generated remains unclear. Secondary structures formed during replication or transcription are vulnerable to oxidative damage and the most common oxidation product is 7,8-dihydro-8-oxoguanine (8-oxoG) (Jarem et al., 2011). As a result, base excision repair (BER) of 8-oxoG results in strand displacement synthesis due to polymerase slippage, resulting in the formation of repeat-associated hairpins on either the lesion or the opposite non-lesion strand (Lokanga et al., 2015) (Figure 3C). Therefore, repairing one lesion increases the possibility of generating additional oxidized bases and cycle repeat instability (Jarem et al., 2011). The observation that the treatment of FXD mouse models with potassium bromate (KBrO3) resulted in a significant increase in both 8-oxoG and the frequency of germline expansion supports the role of oxidative damage in CGG repeat expansion (Entezam et al., 2010). However, this study did not provide any evidence of somatic expansion. MutLγ function is partly dependent on cytosine deamination and AP endonuclease 1 (Apn1) activity, which act on dsDNA (Su and Freudenreich, 2017; Zhao et al., 2018). Therefore, R-loop displacement may act as a substrate for MutLγ, resulting in a slipped strand structure with hairpins on both strands (Reddy et al., 2014). Moreover, in FXS, MutLγ recognizes hairpin junctions as Holliday junctions, nicks both strands, and results in a double stranded break (DSB) in the CGG repeats (Gazy et al., 2019). In addition, a nick can direct MutLγ endonuclease activity to cleave the opposite strand in a concerted manner to generate DSBs (Figure 3D). A recent study has reported that FXS cells show more DSBs that colocalize with R-loop-forming sequences. These R-loop-induced DSBs decrease in number once exogenous FMRP is expressed in FXS cells, suggesting that FMRP prevents the gene from forming an R-loop (Chakraborty et al., 2021).

### CGG/CCG repeat associated secondary structures play a role in the pathogenesis of FXTAS

**FIGURE 4:**
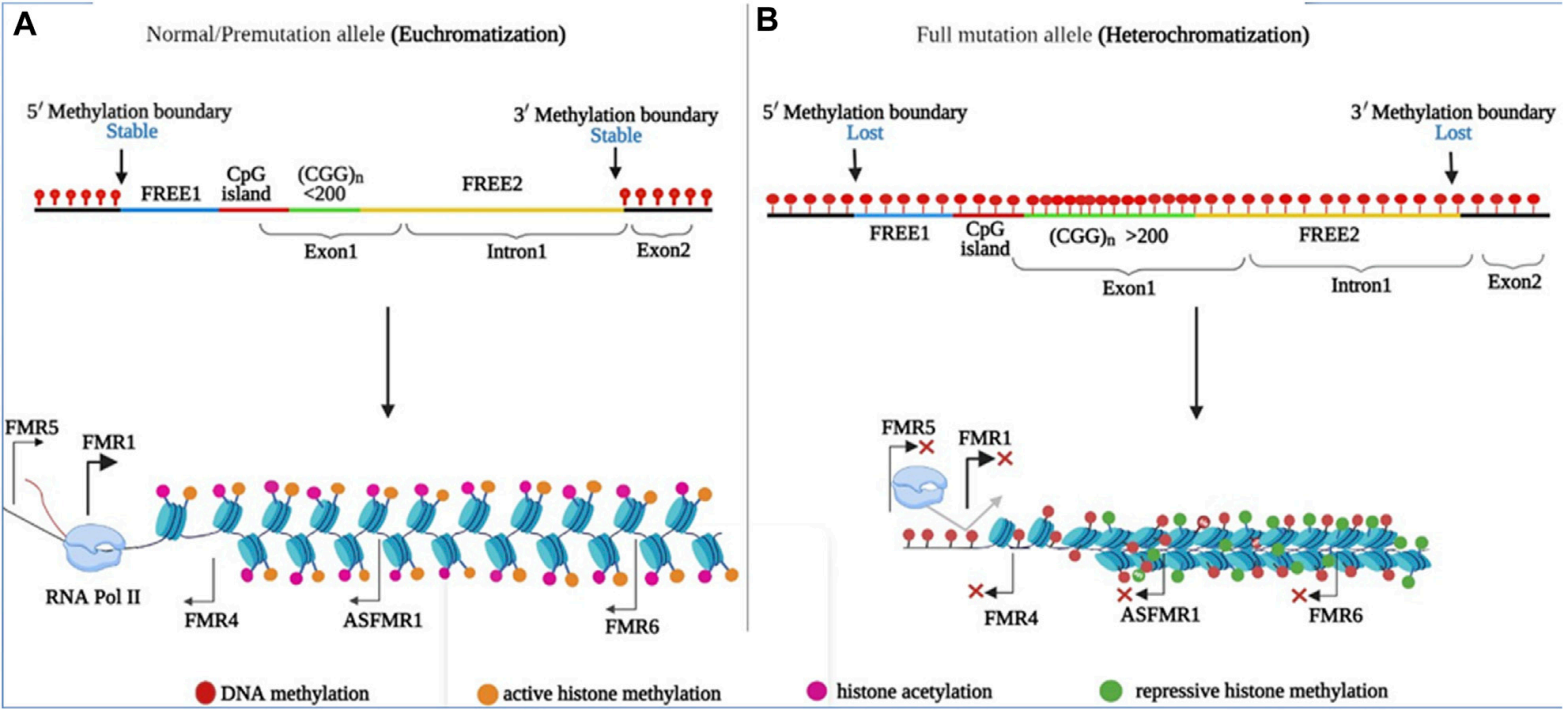
Regions and epigenetic modifications in the FMR1 promoter are shown. (FREE1 region (blue), CpG island (red), CGG repeat (yellow), exon 1, and FREE2 intron 1 segment are highlighted (yellow). **(A)** In normal and PM alleles, the CGG repeats in the promoter region are flanked by 5′ and 3′ stable epigenetic boundaries (DNA methylation (lower) and repressive histone marks (lower), allowing transcription of the FMR1, ASFMR1, FMR4, FMR5, and FMR6 genes. **(B)** The 5′ and 3′ epigenetic boundaries were abolished in FM, allowing DNA methylation to spread throughout the promoter region. DNA methylation (higher) and repressive histone marks (higher).

**FIGURE 5:**
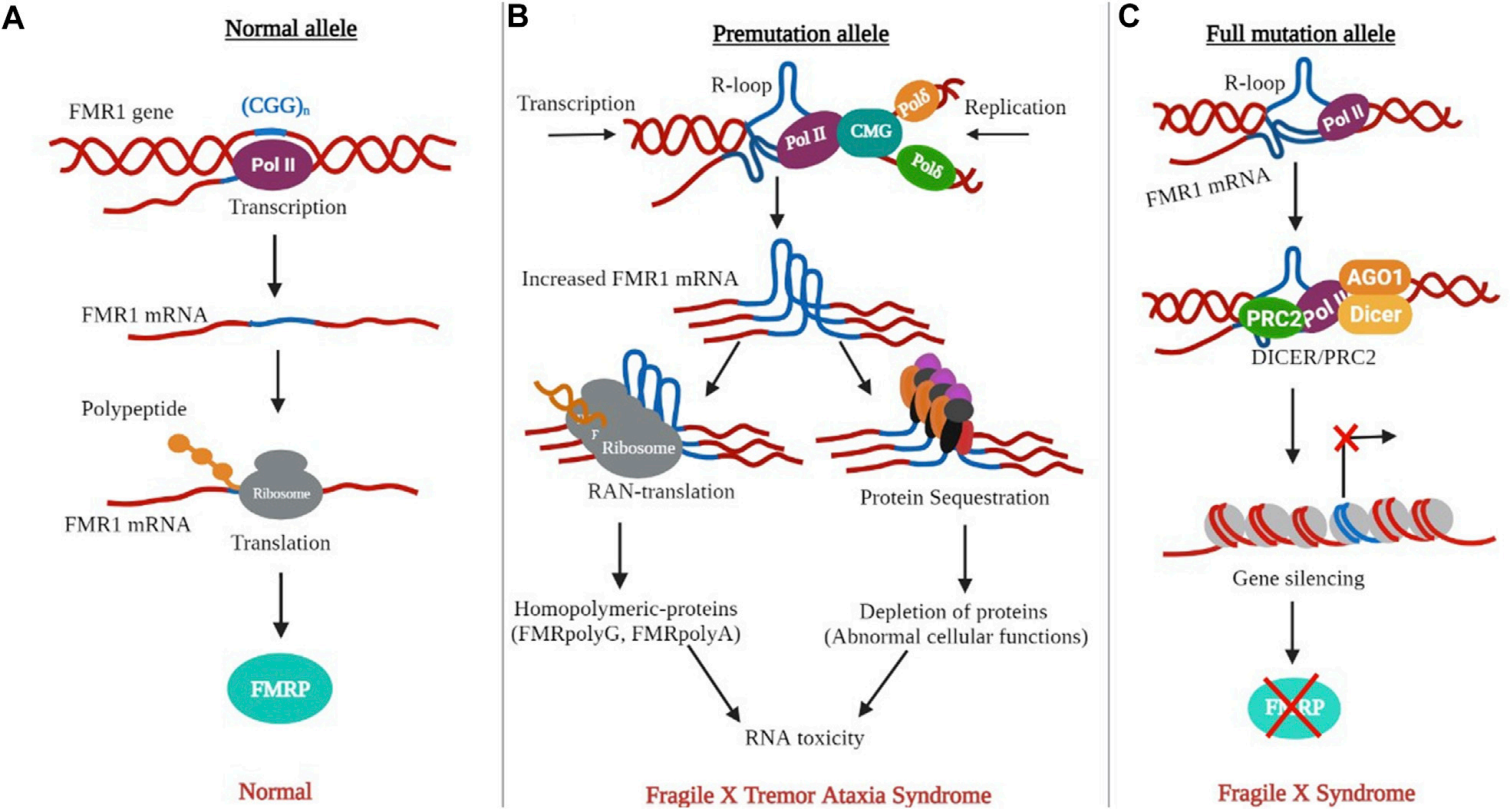
The non-canonical DNA and RNA structures are linked to FXTAS and FXS. **(A)** The presence of normal alleles results in normal transcription and FMRP synthesis. **(B)** The PM allele causes the formation of R-loops in DNA and hairpins (in DNA or RNA). Hairpin-containing FMR1 transcripts can bind and sequester rCGG specific RBPs or induce RAN translation. **(C)** The development of a longer R-loop permits the recruitment of PRC2 to the promoter for repressive histone modification.

Normal FMR1 alleles are transcriptionally active and are correlated with normal FMRP production (Figure 5A). The PM allele is associated with euchromatization and transcriptional activation of the FMR1 gene in PM-related disorders, such as FXTAS and FXPOI. This was evidenced by the increased levels of CGG-containing FMR1 mRNA (up to eight-fold) with relatively unchanged or slightly reduced FMRP levels in PM carriers. FMR1 RNA transcripts are present in the nuclear inclusions (NIs) of postmortem FXTAS brains (Tassone et al., 2004). Related inclusions were found in FXTAS disease model systems. Although higher RNA levels are associated with increased transcription initiation, rather than increased transcript stability (Tassone et al., 2007), the exact mechanism of hyperexpression remains unknown. Several points of evidence may explain hyperexpression of the PM allele. First, both *in vitro* and *in vivo* studies have linked PM alleles, as well as long tracts of CGG/CCG repeats, to a transcriptionally active euchromatic configuration of the FMR1 locus (Figure 4A). This may increase the accessibility of transcription factors or chromatin modifiers to promote transcription initiation. Consistent with this, the FMR1 promoter in PM alleles showed almost two times higher acetylation of histone-H3 and -H4 compared to normal alleles (Todd et al., 2010). Secondly, FMR1 mRNAs with CCG repeats in the PM range form hairpin structures. These structures may directly bind to factors that remodel chromatin to regulate FMR1 transcription or cause stalling of the 40 S ribosomal subunits, resulting in altered transcription start sites and decreased FMRP levels (Usdin and Woodford, 1995). Third, unlike the stable R-loops found in FXS, R-loops associated with PM alleles are susceptible to chromatin decondensation (Wang et al., 1996; Wang, 2007; Powell et al., 2013). As nascent FMR1 and R-loops have been identified as targets of DNA methyltransferase 1 (DNMT1), nascent FMR1 RNA and co-transcriptional R-loop structures may interact with DNMT1, preventing it from performing normal DNA methylation at the FMR1 locus (Di Ruscio et al., 2013). The absence of FXTAS symptoms in FXS patients and the absence of FXS in older FXTAS patients suggests that FMR1 mRNA repeats play a direct role in FXTAS pathology. In model systems, ectopic expression of riboCGG repeats leads to the production of inclusions, disruption of the nuclear lamin A/C architecture, and induction of cell toxicity (Hagerman, 2013). Several mutually non-exclusive molecular mechanisms have been proposed for FXTAS (Figure 5B): RNA gain of function or sequestration type of mechanism has been proposed for REDs such as spinocerebellar ataxia type 8 (SCA8), as well as myotonic dystrophy type 1 (DM1) (La Spada and Taylor, 2010; Todd and Paulson, 2010). According to this model, cellular toxicity is caused by partial sequestration of specific RNA-binding proteins (RBPs) from their normal functions by hairpin structures (Figure 5B). Some of the sequestered proteins identified in FXTAS patients and model systems include heterogeneous nuclear ribonucleoproteins (hnRNP A2/B) and Pur α (Jin et al., 2007; Sofola et al., 2007; Hagerman and Hagerman, 2016), which are involved in various processes of DNA metabolism, including transcriptional activation. Sequestration of muscleblind-like splicing regulator 1 (MBNL1) and SRC associated mitosis of 68 kDa (Sam68) are involved in mRNA splicing defects in FXTAS cellular models (Sellier et al., 2010). Similarly, the sequestration of Drosha and DiGeorge syndrome critical region 8 complex (Drosha-DGCR8) is involved in the processing of miRNA precursors in the nucleus (Sellier et al., 2013) and has been linked to the reduced generation of mature miRNAs in the brains of FXTAS patients. Moreover, overexpression of most RBPs has been shown to reduce RNA toxicity and improve phenotypes in FXTAS disease models (Hagerman, 2012). Recently, it has been found that various DNA helicases, such as human DNA helicase B, remove CGG repeat-associated secondary structures by unwinding (Guler et al., 2018). Consistent with this, R-loop formation can be prevented by RNA helicases, as overexpression of the *Drosophila* ortholog of p68/DDX5 RNA helicase, Rm62 (one of the sequestered proteins along with Pur α), prevents neurodegeneration in transgenic flies expressing riboCGG repeats within the PM range (Qurashi et al., 2011). Another proposed mechanism for FXTAS pathogenesis is RAN translation, which is thought to be triggered by RNA hairpins acting as impediments to ribosomes that favor noncanonical translation at suboptimal initiation codons upstream of the true initiation codon. In FXTAS, the non-coding region of FMR1 mRNA is translated into multiple RAN translation products, including homopolymeric proteins such as FMRpolyG, whose length correlates with the number of CGG repeats (Todd et al., 2013). RAN translation has been detected in several other REDs such as amyotrophic lateral sclerosis, frontal dementia (ALS-FTD), and SCA8, suggesting shared disease mechanisms (Cleary and Ranum, 2013). The FMRpolyG peptide was found in ubiquitin-positive inclusions in the brains of FXTAS patients, and has been directly linked to CGG repeat-associated toxicity in FXTAS disease models (Todd et al., 2013; Buijsen et al., 2014; Sellier et al., 2017). FMRpolyG binds to CGG-RNA quadruplex structures *in vitro*, promotes aggregate formation, and alters the ubiquitin-proteasome system (UPS) in an FXTAS model system. In addition, FMRpolyG interacts with lamina-associated polypeptide 2 beta (LAP2β), a nuclear membrane protein, and rescues neuronal cell death in a mouse FXTAS model (Sellier et al., 2013; Todd et al., 2013; Hoem et al., 2019). The third proposed molecular mechanism involves an altered DNA damage response (DDR) molecular signalling pathway due to co-transcriptional R-loops (Aguilera and García-Muse, 2012; García-Muse and Loops, 2019). Such R-loops are susceptible to single- and double-strand breaks (Cristini et al., 2019). Corroborating this, γH2AX, a marker of DSBs, has been identified in NIs in FXTAS brains (Iwahashi et al., 2006; Garcia-Arocena and Hagerman, 2010; Hoem et al., 2011). Similarly, DSB-activated ataxia-telangiectasia mutated kinase (ATM) has been observed in FXTAS animal models (Robin et al., 2017).

### CGG/CCG repeat associated secondary structures play a role in the pathogenesis of FXS

The transcriptionally inactive FM allele is linked to the heterochromatic status of FMR1, as has been observed in individuals with FXS. During embryonic development, *de novo* DNA methyltransferases (DNMTs) establish cytosine methylation across the entire promoter, including the fragile X related element 1 (FREE1), CpG island, CGG repeat, and fragile X related element 2 (FREE2) regions of FMR1 gene (Oberlé et al., 1991; Naumann et al., 2009). However, in rare FXS individuals, the unmethylated full mutation (UFM) allele may represent the methylation status prior to FMR1 silencing, which occurs around 11 weeks of gestation (Willemsen et al., 2002; Colak et al., 2014; Mor-Shaked and Eiges, 2018). Thus, the extent to which silencing occurs in early FXS embryos remains an important open question. In addition, FMR1 silencing may require several other epigenetic regulatory mechanisms. Histone modifications occur in FMR1 promoter-associated chromatin, with inhibitory histone marks (H3K9me2, H3K9me3, H3K27me3, and H4K20me3) and fewer active histone marks (H3K9ac and H4K16ac) catalyzed by histone methyltransferase (HMT) and histone deacetylases, respectively (Figure 4B) (Coffee et al., 1999; Biacsi et al., 2008; Li et al., 2018). Polycomb group proteins cause the trimethylation of histone 3, such as H3K9me3, H3K27me3, and H4K20me3. Specifically, polycomb repressive complex 2 (PRC2), a transcriptional repressor complex, is required for histone 3 trimethylation at lysine 27 (H3K27me3), which is a late modification required for gene silencing. Consequently, PRC2 inhibition prevents H3K27me3 in the FMR1 5′-UTR (Kumari and Usdin, 2014). PRC2 binds to G-rich RNAs, specifically G4-forming RNA sequences and R-loops, to mediate gene silencing at multiple loci (Skourti-Stathaki et al., 2019). Therefore, it is possible that the R-loops and FMR1 transcript aid gene silencing by facilitating PRC2 recruitment, either directly or indirectly (Figure 5C). Accordingly, FMR1 mRNA, and thus R-loops prevents PRC2 mediated gene silencing during the neuronal differentiation of embryonic stem cells (Colak et al., 2014). In addition, decreased PRC2 recruitment to FM alleles is reactivated by 5-azadeoxycytidine (Kumari and Usdin, 2014). It is worth noting that given the proposed role of R-loops in hyperexpression of the PM allele, the role of the R-loop in gene silencing in the case of the FM allele appears paradoxical. The R loops associated with FM alleles are more stable and longer, which may account for the differences in the effects of repeat length, transcriptional rate, protein expression, and cell stage (Colak et al., 2014; Groh and Gromak, 2014; Loomis et al., 2014). As a result, this R-loop may further promote the loss of active chromatin marks in the flanking regions of the FMR1 promoter, transcriptional termination, and DNA damage.

### Novel CGG/CCG repeats in the human genome suggest their broad involvement in neurological diseases

Long-read and whole-genome sequencing has revealed additional STRs within the genome that are more widespread than previously thought (Depienne and Mandel, 2021). A small subset of these STRs has identical sequences, sizes, and genomic locations. In addition, they may be unstable during intergenerational transmission and exhibit expansions or contractions that result in neurological disorders with related clinical manifestations and pathogenic mechanisms (Liufu et al., 2022). For example, similar to FXS, expanded CGG repeats are a causative genetic contributor to Desbuquois dysplasia 2 (DBQD2) and Baratela-Scott syndrome (BSS). DBQD2 and BSS are characterised by skeletal dysplasia and share several clinical features. In both cases, CGG expansion in the 5′-UTR of XYLT1 leads to gene silencing through hypermethylation (LaCroix et al., 2019). Similarly, hypermethylation caused by CGG expansion in the 5′-UTR of disco-interacting protein 2 homologue B (DIP2B) (Winnepenninckx et al., 2007) and AF4/FMR2 family member 3 (AFF3) causes FRA12A-related neurocognitive and ID disorders (Knight et al., 1993). Similar clinical manifestations have been observed in individuals with deletions or other loss-of-function mutations in these genes, further supporting the hypothesis that CGG expansion in these genes is pathogenic *via* a loss-of-function mechanism.

CGG expansion in several other genes can also manifest as dominant neurodegenerative disorders *via* mechanisms similar to those described for FXTAS. The GGC repeat, located in the 5′-UTR of NOTCH2NLC, is a causative genetic contributor to neuronal intranuclear inclusion disease (NIID) (Deng et al., 2019; Ishiura et al., 2019). Pathogenic NOTCH2NLC expansions have been identified in patients with essential tremor (ETM6, MIM #618866), C9ORF72-associated amyotrophic lateral sclerosis/frontal temporal dementia (ALS/FTD) (Tian et al., 2019; Jiao et al., 2020), Parkinsonism (Ma et al., 2020), and multiple system atrophy (Fang et al., 2020). In addition, oculopharyngodistal myopathy type 1–4 (OPDM), group of adult-onset inherited neuromuscular disorders, are caused by CGG repeat expansions in the 5′UTR of LRP12 (Ishiura et al., 2019), GIPC1 (Deng et al., 2020), NOTCH2NLC (Yu et al., 2021), and RILPL1 (Yu et al., 2022), respectively. Similarly, CGG expansion in NUTM2B-AS1 has been identified as the causative agent of oculopharyngeal myopathy with leucoencephalopathy (OPML) (Ishiura et al., 2019). Interestingly, NIID, OPDM, and OPML resemble FXTAS in terms of clinical symptoms, radiological imaging, and histological characteristics, such as the presence of distinctive eosinophilic ubiquitin-positive NIs (Viguera et al., 2001). In patients with NIID, RNA molecules with expanded CGG repeats form RNA foci that sequester RBPs into p62-positive NIs (Mori et al., 2012). In addition, similar to FXTAS patients, the translation of expanded GGC repeats resulted in the accumulation of polyG-containing proteins in the NIs in both the NIID model system and patients. Together, these results suggest a pathological mechanism involving toxic gain-of-function at the RNA level and/or RAN translation. Although the formation of polyG in OPML and OPDMs has not yet been elucidated, in the C9ORF72-associated amyotrophic ALS/FTD, translation of the polyglycine-alanine dipeptide repeat (polyGA DPR) protein occurs because of G4C2 repeats located in the first intron of the C9ORF72 gene (Tabet et al., 2018). While these examples demonstrate common pathogenic mechanisms in several distinct diseases, it remains unclear whether they reflect general disease mechanisms. It is worth noting that DNA methylation may be protective in some NOTCH2NLC-associated NIIDs (Ishiura and Tsuji, 2020), however, it increases RNA and peptide toxicity in C9ORF72-associated ALS/FTD (Zhu et al., 2020). Therefore, understanding how DNA methylation affects the progression of such disorders can lead to improved treatments such as those based on Cas9 methylation editing, which has recently been proposed for FXS (Liu et al., 2018).

## Conclusion and perspective

FXTAS and FXS are two primary diseases caused by dynamic mutations in FMR1, and have distinct clinical manifestations and molecular pathogenesis. FXTAS is a late-onset neurodegenerative disorder that typically affects men >50 years of age. On the other hand, FXS is a neurodevelopmental disease and the most common type of inherited intellectual disability. Both are caused by the abnormal expansion of CGG repeats beyond the normal range in the 5′-UTR of the FMR1 gene. PM alleles (CGG expansions of 55–200 repeats) were associated with elevated FMR1 mRNA levels and relatively normal FMRP levels. In contrast, FM alleles (CGG expansion of ≥200 repeats) typically result in transcriptional silencing and, consequently, the loss of FMR1 protein (FMRP). Abnormal CGG expansions form a variety of secondary structures that are linked to the pathology and transmission risk in both diseases. As CGG/CCG/GGC repeats with characteristics similar to those of CGG repeat expansions associated with FXS or FXTAS are abundant in the human genome, these studies suggest that CGG repeats are broadly involved in neurological diseases. Although several rare folate-sensitive fragile sites associated with neurodevelopmental diseases have been cloned as expanded CGG repeats, the number of studies of CGG/GGC repeat-related disorders has increased in recent years. A recent study using whole-genome STR analysis discovered hundreds of unique-CGG repeats with highly variable repeat lengths and intergenerational instability, most of which are linked to known neurodevelopmental disease genes or strong candidate genes (Annear et al., 2021). Furthermore, several GGC repeat-related disorders, such as ET and NIID, have been identified to have clinical and molecular overlaps with FXTAS (Xu et al., 2021). In these diseases, GGC repeats occur at the 5′-UTR of the respective gene and do not involve the open reading frame of the gene, implying that GGC and CGG repeat RNAs share the same secondary structures that may play an important role in disease pathogenesis and are thus amenable to pharmacological or molecular therapy. In this context, antisense oligonucleotides (ASOs) containing CCG repeats have been shown to reduce R-loop formation and alleviate the downstream effects of RNA hairpin formation (Derbis et al., 2021). Similarly, small molecules that inhibit protein binding to the hairpin structure or reduce RBP sequestration or RAN translation have been shown to alleviate disease pathology in FXTAS model systems (Disney et al., 2012; Hagihara et al., 2012; Qurashi et al., 2012; Tran et al., 2014; Verma et al., 2019; Verma et al., 2020). Despite these encouraging results, a deeper understanding of the underlying pathophysiology of these diseases is still required. Recently, small molecules that reprogram the epigenetically determined transcriptional state of key genes by stabilizing G4 structures in DNA have been used to develop epigenetic therapies (Guilbaud et al., 2017). Therefore, understanding the secondary structures formed by CGG/GGC repeats and their downstream effects may lead to a better understanding of disease pathology as well as the development of therapeutics to alleviate their pathological effects.

## References

[B1] Abu Diab M. Mor-Shaked H. Cohen E. Cohen-Hadad Y. Ram O. Epsztejn-Litman S. (2018). The G-rich repeats in FMR1 and C9orf72 loci are hotspots for local unpairing of DNA. Genetics 210 (4), 1239–1252. Epub 20181105PubMed PMID: 30396881; PubMed Central PMCID: PMC6283162. 10.1534/genetics.118.301672 30396881 PMC6283162

[B2] Aguilera A. García-Muse T. (2012). R loops: From transcription byproducts to threats to genome stability. Mol. Cell 46 (2), 115–124. PubMed PMID: 22541554. 10.1016/j.molcel.2012.04.009 22541554

[B3] Ajjugal Y. Kolimi N. Rathinavelan T. (2021). Secondary structural choice of DNA and RNA associated with CGG/CCG trinucleotide repeat expansion rationalizes the RNA misprocessing in FXTAS. Sci. Rep. 11 (1), 8163. Epub 20210414PubMed PMID: 33854084; PubMed Central PMCID: PMC8046799. 10.1038/s41598-021-87097-y 33854084 PMC8046799

[B4] Annear D. J. Vandeweyer G. Elinck E. Sanchis-Juan A. French C. E. Raymond L. (2021). Abundancy of polymorphic CGG repeats in the human genome suggest a broad involvement in neurological disease. Sci. Rep. 11 (1), 2515. Epub 20210128PubMed PMID: 33510257; PubMed Central PMCID: PMC7844047. 10.1038/s41598-021-82050-5 33510257 PMC7844047

[B5] Asamitsu S. Yabuki Y. Ikenoshita S. Kawakubo K. Kawasaki M. Usuki S. (2021). CGG repeat RNA G-quadruplexes interact with FMRpolyG to cause neuronal dysfunction in fragile X-related tremor/ataxia syndrome. Sci. Adv. 7 (3), eabd9440. Epub 20210113PubMed PMID: 33523882; PubMed Central PMCID: PMC7806243. 10.1126/sciadv.abd9440 33523882 PMC7806243

[B6] Bagshaw A. T. M. (2017). Functional mechanisms of microsatellite DNA in eukaryotic genomes. Genome Biol. Evol. 9 (9), 2428–2443. PubMed PMID: 28957459; PubMed Central PMCID: PMC5622345. 10.1093/gbe/evx164 28957459 PMC5622345

[B7] Belotserkovskii B. P. Neil A. J. Saleh S. S. Shin J. H. Mirkin S. M. Hanawalt P. C. (2013). Transcription blockage by homopurine DNA sequences: Role of sequence composition and single-strand breaks. Nucleic Acids Res. 41 (3), 1817–1828. Epub 20121228PubMed PMID: 23275544; PubMed Central PMCID: PMC3561996. 10.1093/nar/gks1333 23275544 PMC3561996

[B8] Bhat S. A. Yousuf A. Mushtaq Z. Kumar V. Qurashi A. (2021). Fragile X premutation rCGG repeats impair synaptic growth and synaptic transmission at Drosophila larval neuromuscular junction. Hum. Mol. Genet. 30 (18), 1677–1692. PubMed PMID: 33772546. 10.1093/hmg/ddab087 33772546

[B9] Biacsi R. Kumari D. Usdin K. (2008). SIRT1 inhibition alleviates gene silencing in Fragile X mental retardation syndrome. PLoS Genet. 4 (3), e1000017. Epub 20080307PubMed PMID: 18369442; PubMed Central PMCID: PMC2265469. 10.1371/journal.pgen.1000017 18369442 PMC2265469

[B10] Buijsen R. A. Sellier C. Severijnen L. A. Oulad-Abdelghani M. Verhagen R. F. Berman R. F. (2014). FMRpolyG-positive inclusions in CNS and non-CNS organs of a fragile X premutation carrier with fragile X-associated tremor/ataxia syndrome. Acta Neuropathol. Commun. 2, 162. Epub 20141126PubMed PMID: 25471011; PubMed Central PMCID: PMC4254384. 10.1186/s40478-014-0162-2 25471011 PMC4254384

[B11] Chakraborty A. Jenjaroenpun P. Li J. El Hilali S. McCulley A. Haarer B. (2021). Replication stress induces global chromosome breakage in the fragile X genome. Cell Rep. 34 (12), 108179. PubMed PMID: 33761342; PubMed Central PMCID: PMC8045960. 10.1016/j.celrep.2020.108179 PMC8045960 33761342

[B12] Chen X. Mariappan S. V. Catasti P. Ratliff R. Moyzis R. K. Laayoun A. (1995). Hairpins are formed by the single DNA strands of the fragile X triplet repeats: Structure and biological implications. Proc. Natl. Acad. Sci. U. S. A. 92 (11), 5199–5203. PubMed PMID: 7761473; PubMed Central PMCID: PMC41876. 10.1073/pnas.92.11.5199 7761473 PMC41876

[B13] Chen Y. W. Satange R. Wu P. C. Jhan C. R. Chang C. K. Chung K. R. (2018). CoII(Chromomycin)₂ complex induces a conformational change of CCG repeats from i-motif to base-extruded DNA duplex. Int. J. Mol. Sci. 19 (9), E2796. Epub 20180917PubMed PMID: 30227633; PubMed Central PMCID: PMC6164834. 10.3390/ijms19092796 30227633 PMC6164834

[B14] Ciesiolka A. Jazurek M. Drazkowska K. Krzyzosiak W. J. (2017). Structural characteristics of simple RNA repeats associated with disease and their deleterious protein interactions. Front. Cell. Neurosci. 11, 97. Epub 20170411PubMed PMID: 28442996; PubMed Central PMCID: PMC5387085. 10.3389/fncel.2017.00097 28442996 PMC5387085

[B15] Cleary J. D. Ranum L. P. (2013). Repeat-associated non-ATG (RAN) translation in neurological disease. Hum. Mol. Genet. 22 (R1), R45–R51. Epub 20130804PubMed PMID: 23918658; PubMed Central PMCID: PMC3782068. 10.1093/hmg/ddt371 23918658 PMC3782068

[B16] Coffee B. Zhang F. Warren S. T. Reines D. (1999). Acetylated histones are associated with FMR1 in normal but not fragile X-syndrome cells. Nat. Genet. 22 (1), 98–101. PubMed PMID: 10319871. 10.1038/8807 10319871

[B17] Colak D. Zaninovic N. Cohen M. S. Rosenwaks Z. Yang W. Y. Gerhardt J. (2014). Promoter-bound trinucleotide repeat mRNA drives epigenetic silencing in fragile X syndrome. Science 343 (6174), 1002–1005. PubMed PMID: 24578575; PubMed Central PMCID: PMC4357282. 10.1126/science.1245831 24578575 PMC4357282

[B18] Cristini A. Ricci G. Britton S. Salimbeni S. Huang S. N. Marinello J. (2019). Dual processing of R-loops and topoisomerase I induces transcription-dependent DNA double-strand breaks. Cell Rep. 28 (12), 3167–3181. e6PubMed PMID: 31533039; PubMed Central PMCID: PMC8274950. 10.1016/j.celrep.2019.08.041 31533039 PMC8274950

[B19] Crossley M. P. Bocek M. Cimprich K. A. (2019). R-loops as cellular regulators and genomic threats. Mol. Cell 73 (3), 398–411. PubMed PMID: 30735654; PubMed Central PMCID: PMC6402819. 10.1016/j.molcel.2019.01.024 30735654 PMC6402819

[B20] Deng J. Gu M. Miao Y. Yao S. Zhu M. Fang P. (2019). Long-read sequencing identified repeat expansions in the 5'UTR of the NOTCH2NLC gene from Chinese patients with neuronal intranuclear inclusion disease. J. Med. Genet. 56 (11), 758–764. Epub 20190814PubMed PMID: 31413119. 10.1136/jmedgenet-2019-106268 31413119

[B21] Deng J. Yu J. Li P. Luan X. Cao L. Zhao J. (2020). Expansion of GGC repeat in GIPC1 is associated with oculopharyngodistal myopathy. Am. J. Hum. Genet. 106 (6), 793–804. Epub 20200514PubMed PMID: 32413282; PubMed Central PMCID: PMC7273532. 10.1016/j.ajhg.2020.04.011 32413282 PMC7273532

[B22] Depienne C. Mandel J. L. (2021). 30 years of repeat expansion disorders: What have we learned and what are the remaining challenges? Am. J. Hum. Genet. 108 (5), 764–785. Epub 20210402PubMed PMID: 33811808; PubMed Central PMCID: PMC8205997. 10.1016/j.ajhg.2021.03.011 33811808 PMC8205997

[B23] Derbis M. Kul E. Niewiadomska D. Sekrecki M. Piasecka A. Taylor K. (2021). Short antisense oligonucleotides alleviate the pleiotropic toxicity of RNA harboring expanded CGG repeats. Nat. Commun. 12 (1), 1265. Epub 20210224PubMed PMID: 33627639; PubMed Central PMCID: PMC7904788. 10.1038/s41467-021-21021-w 33627639 PMC7904788

[B24] Di Ruscio A. Ebralidze A. K. Benoukraf T. Amabile G. Goff L. A. Terragni J. (2013). DNMT1-interacting RNAs block gene-specific DNA methylation. Nature 503 (7476), 371–376. Epub 20131009PubMed PMID: 24107992; PubMed Central PMCID: PMC3870304. 10.1038/nature12598 24107992 PMC3870304

[B25] Disney M. D. Liu B. Yang W. Y. Sellier C. Tran T. Charlet-Berguerand N. (2012). A small molecule that targets r(CGG)(exp) and improves defects in fragile X-associated tremor ataxia syndrome. ACS Chem. Biol. 7 (10), 1711–1718. Epub 20120904PubMed PMID: 22948243; PubMed Central PMCID: PMC3477254. 10.1021/cb300135h 22948243 PMC3477254

[B26] Entezam A. Lokanga A. R. Le W. Hoffman G. Usdin K. (2010). Potassium bromate, a potent DNA oxidizing agent, exacerbates germline repeat expansion in a fragile X premutation mouse model. Hum. Mutat. 31 (5), 611–616. PubMed PMID: 20213777; PubMed Central PMCID: PMC2951473. 10.1002/humu.21237 20213777 PMC2951473

[B27] Fang P. Yu Y. Yao S. Chen S. Zhu M. Chen Y. (2020). Repeat expansion scanning of the NOTCH2NLC gene in patients with multiple system atrophy. Ann. Clin. Transl. Neurol. 7 (4), 517–526. Epub 20200406PubMed PMID: 32250060; PubMed Central PMCID: PMC7187708. 10.1002/acn3.51021 32250060 PMC7187708

[B28] Fojtík P. Vorlícková M. (2001). The fragile X chromosome (GCC) repeat folds into a DNA tetraplex at neutral pH. Nucleic Acids Res. 29 (22), 4684–4690. PubMed PMID: 11713318; PubMed Central PMCID: PMC92515. 10.1093/nar/29.22.4684 11713318 PMC92515

[B29] Garcia-Arocena D. Hagerman P. J. (2010). Advances in understanding the molecular basis of FXTAS. Hum. Mol. Genet. 19 (R1), R83–R89. Epub 2010/05/01PubMed PMID: 20430935; PubMed Central PMCID: PMC2875053. 10.1093/hmg/ddq166 20430935 PMC2875053

[B30] García-Muse T. Loops Aguilera A. R. (2019). R loops: From physiological to pathological roles. Cell 179 (3), 604–618. Epub 20191010PubMed PMID: 31607512. 10.1016/j.cell.2019.08.055 31607512

[B31] Gazy I. Hayward B. Potapova S. Zhao X. Usdin K. (2019). Double-strand break repair plays a role in repeat instability in a fragile X mouse model. DNA Repair (Amst) 74, 63–69. Epub 2019/01/05PubMed PMID: 30606610; PubMed Central PMCID: PMC6366319. 10.1016/j.dnarep.2018.12.004 30606610 PMC6366319

[B32] Gerhardt J. Tomishima M. J. Zaninovic N. Colak D. Yan Z. Zhan Q. (2014). The DNA replication program is altered at the FMR1 locus in fragile X embryonic stem cells. Mol. Cell 53 (1), 19–31. Epub 20131127PubMed PMID: 24289922; PubMed Central PMCID: PMC3920742. 10.1016/j.molcel.2013.10.029 24289922 PMC3920742

[B33] Groh M. Gromak N. (2014). Out of balance: R-Loops in human disease. PLoS Genet. 10 (9), e1004630. Epub 20140918PubMed PMID: 25233079; PubMed Central PMCID: PMC4169248. 10.1371/journal.pgen.1004630 25233079 PMC4169248

[B34] Guilbaud G. Murat P. Recolin B. Campbell B. C. Maiter A. Sale J. E. (2017). Local epigenetic reprogramming induced by G-quadruplex ligands. Nat. Chem. 9 (11), 1110–1117. Epub 20170731PubMed PMID: 29064488; PubMed Central PMCID: PMC5669467. 10.1038/nchem.2828 29064488 PMC5669467

[B35] Guler G. D. Rosenwaks Z. Gerhardt J. (2018). Human DNA helicase B as a candidate for unwinding secondary CGG repeat structures at the fragile X mental retardation gene. Front. Mol. Neurosci. 11, 138. Epub 20180430PubMed PMID: 29760651; PubMed Central PMCID: PMC5936766. 10.3389/fnmol.2018.00138 29760651 PMC5936766

[B36] Hagerman P. (2013). Fragile X-associated tremor/ataxia syndrome (FXTAS): Pathology and mechanisms. Acta Neuropathol. 126 (1), 1–19. Epub 2013/06/26PubMed PMID: 23793382; PubMed Central PMCID: PMC3904666. 10.1007/s00401-013-1138-1 23793382 PMC3904666

[B37] Hagerman P. J. (2012). Current gaps in understanding the molecular basis of FXTAS. Tremor Other Hyperkinet Mov. (N Y) 2. Epub 2013/02/27PubMed PMID: 23440729; PubMed Central PMCID: PMC3379894. 10.7916/d80c4th0 PMC3379894 23440729

[B38] Hagerman P. J. Hagerman R. J. (2004). The fragile-X premutation: A maturing perspective. Am. J. Hum. Genet. 74 (5), 805–816. Epub 20040329PubMed PMID: 15052536; PubMed Central PMCID: PMC1181976. 10.1086/386296 15052536 PMC1181976

[B39] Hagerman R. Hoem G. Hagerman P. (2010). Fragile X and autism: Intertwined at the molecular level leading to targeted treatments. Mol. Autism 1 (1), 12. Epub 2010/09/21PubMed PMID: 20858229; PubMed Central PMCID: PMC2954865. 10.1186/2040-2392-1-12 20858229 PMC2954865

[B40] Hagerman R. J. Hagerman P. (2016). Fragile X-associated tremor/ataxia syndrome - features, mechanisms and management. Nat. Rev. Neurol. 12 (7), 403–412. Epub 2016/06/25PubMed PMID: 27340021. 10.1038/nrneurol.2016.82 27340021

[B41] Hagerman R. J. Protic D. Rajaratnam A. Salcedo-Arellano M. J. Aydin E. Y. Schneider A. (2018). Fragile X-associated neuropsychiatric disorders (FXAND). Front. Psychiatry 9, 564. Epub 2018/11/30PubMed PMID: 30483160; PubMed Central PMCID: PMC6243096. 10.3389/fpsyt.2018.00564 30483160 PMC6243096

[B42] Hagihara M. He H. Kimura M. Nakatani K. (2012). A small molecule regulates hairpin structures in d(CGG) trinucleotide repeats. Bioorg. Med. Chem. Lett. 22 (5), 2000–2003. Epub 20120125PubMed PMID: 22326165. 10.1016/j.bmcl.2012.01.030 22326165

[B43] Handa V. Saha T. Usdin K. (2003). The fragile X syndrome repeats form RNA hairpins that do not activate the interferon-inducible protein kinase, PKR, but are cut by Dicer. Nucleic Acids Res. 31 (21), 6243–6248. PubMed PMID: 14576312; PubMed Central PMCID: PMC275460. 10.1093/nar/gkg818 14576312 PMC275460

[B44] Hayward B. E. Steinbach P. J. Usdin K. (2020). A point mutation in the nuclease domain of MLH3 eliminates repeat expansions in a mouse stem cell model of the Fragile X-related disorders. Nucleic Acids Res. 48 (14), 7856–7863. PubMed PMID: 32619224; PubMed Central PMCID: PMC7430641. 10.1093/nar/gkaa573 32619224 PMC7430641

[B45] Hoem G. Bowitz Larsen K. Overvatn A. Brech A. Lamark T. Sjottem E. (2019). The FMRpolyGlycine protein mediates aggregate formation and toxicity independent of the CGG mRNA hairpin in a cellular model for FXTAS. Front. Genet. 10, 249. Epub 2019/04/16PubMed PMID: 30984240; PubMed Central PMCID: PMC6447689. 10.3389/fgene.2019.00249 30984240 PMC6447689

[B46] Hoem G. Raske C. R. Garcia-Arocena D. Tassone F. Sanchez E. Ludwig A. L. (2011). CGG-repeat length threshold for FMR1 RNA pathogenesis in a cellular model for FXTAS. Hum. Mol. Genet. 20 (11), 2161–2170. Epub 20110309PubMed PMID: 21389081; PubMed Central PMCID: PMC3090194. 10.1093/hmg/ddr101 21389081 PMC3090194

[B47] Ishiura H. Shibata S. Yoshimura J. Suzuki Y. Qu W. Doi K. (2019). Noncoding CGG repeat expansions in neuronal intranuclear inclusion disease, oculopharyngodistal myopathy and an overlapping disease. Nat. Genet. 51 (8), 1222–1232. Epub 2019/07/25PubMed PMID: 31332380. 10.1038/s41588-019-0458-z 31332380

[B48] Ishiura H. Tsuji S. (2020). Advances in repeat expansion diseases and a new concept of repeat motif-phenotype correlation. Curr. Opin. Genet. Dev. 65, 176–185. Epub 20200807PubMed PMID: 32777681. 10.1016/j.gde.2020.05.029 32777681

[B49] Iwahashi C. K. Yasui D. H. An H. J. Greco C. M. Tassone F. Nannen K. (2006). Protein composition of the intranuclear inclusions of FXTAS. Brain 129 (1), 256–271. Epub 2005/10/26PubMed PMID: 16246864. 10.1093/brain/awh650 16246864

[B50] Jarem D. A. Huckaby L. V. Delaney S. (2010). AGG interruptions in (CGG)(n) DNA repeat tracts modulate the structure and thermodynamics of non-B conformations *in vitro* . Biochemistry 49 (32), 6826–6837. PubMed PMID: 20695523; PubMed Central PMCID: PMC3650493. 10.1021/bi1007782 20695523 PMC3650493

[B51] Jarem D. A. Wilson N. R. Schermerhorn K. M. Delaney S. (2011). Incidence and persistence of 8-oxo-7, 8-dihydroguanine within a hairpin intermediate exacerbates a toxic oxidation cycle associated with trinucleotide repeat expansion. DNA Repair (Amst) 10 (8), 887–896. Epub 20110702PubMed PMID: 21727036; PubMed Central PMCID: PMC3146575. 10.1016/j.dnarep.2011.06.003 21727036 PMC3146575

[B52] Jiao B. Zhou L. Zhou Y. Weng L. Liao X. Tian Y. (2020). Identification of expanded repeats in NOTCH2NLC in neurodegenerative dementias. Neurobiol. Aging 89, e1. e7. Epub 20200124PubMed PMID: 32081467. 10.1016/j.neurobiolaging.2020.01.010 32081467

[B53] Jin P. Duan R. Qurashi A. Qin Y. Tian D. Rosser T. C. (2007). Pur alpha binds to rCGG repeats and modulates repeat-mediated neurodegeneration in a Drosophila model of fragile X tremor/ataxia syndrome. Neuron 55 (4), 556–564. PubMed PMID: 17698009; PubMed Central PMCID: PMC1994817. 10.1016/j.neuron.2007.07.020 17698009 PMC1994817

[B54] Kadyrova L. Y. Gujar V. Burdett V. Modrich P. L. Kadyrov F. A. (2020). Human MutLγ, the MLH1-MLH3 heterodimer, is an endonuclease that promotes DNA expansion. Proc. Natl. Acad. Sci. U. S. A. 117 (7), 3535–3542. Epub 20200203PubMed PMID: 32015124; PubMed Central PMCID: PMC7035508. 10.1073/pnas.1914718117 32015124 PMC7035508

[B55] Kearse M. G. Green K. M. Krans A. Rodriguez C. M. Linsalata A. E. Goldstrohm A. C. (2016). CGG repeat-associated non-AUG translation utilizes a cap-dependent scanning mechanism of initiation to produce toxic proteins. Mol. Cell 62 (2), 314–322. Epub 20160331PubMed PMID: 27041225; PubMed Central PMCID: PMC4854189. 10.1016/j.molcel.2016.02.034 27041225 PMC4854189

[B56] Kettani A. Kumar R. A. Patel D. J. (1995). Solution structure of a DNA quadruplex containing the fragile X syndrome triplet repeat. J. Mol. Biol. 254 (4), 638–656. PubMed PMID: 7500339. 10.1006/jmbi.1995.0644 7500339

[B57] Khateb S. Weisman-Shomer P. Hershco-Shani I. Ludwig A. L. Fry M. (2007). The tetraplex (CGG)n destabilizing proteins hnRNP A2 and CBF-A enhance the *in vivo* translation of fragile X premutation mRNA. Nucleic Acids Res. 35 (17), 5775–5788. Epub 20070823PubMed PMID: 17716999; PubMed Central PMCID: PMC2034458. 10.1093/nar/gkm636 17716999 PMC2034458

[B58] Kim J. C. Mirkin S. M. (2013). The balancing act of DNA repeat expansions. Curr. Opin. Genet. Dev. 23 (3), 280–288. Epub 20130529PubMed PMID: 23725800; PubMed Central PMCID: PMC3703482. 10.1016/j.gde.2013.04.009 23725800 PMC3703482

[B59] Knight S. J. Flannery A. V. Hirst M. C. Campbell L. Christodoulou Z. Phelps S. R. (1993). Trinucleotide repeat amplification and hypermethylation of a CpG island in FRAXE mental retardation. Cell 74 (1), 127–134. PubMed PMID: 8334699. 10.1016/0092-8674(93)90300-f 8334699

[B60] Krzyzosiak W. J. Sobczak K. Wojciechowska M. Fiszer A. Mykowska A. Kozlowski P. (2012). Triplet repeat RNA structure and its role as pathogenic agent and therapeutic target. Nucleic Acids Res. 40 (1), 11–26. Epub 20110909PubMed PMID: 21908410; PubMed Central PMCID: PMC3245940. 10.1093/nar/gkr729 21908410 PMC3245940

[B61] Kumari D. Usdin K. (2014). Polycomb group complexes are recruited to reactivated FMR1 alleles in Fragile X syndrome in response to FMR1 transcription. Hum. Mol. Genet. 23 (24), 6575–6583. Epub 20140723PubMed PMID: 25055869; PubMed Central PMCID: PMC4240206. 10.1093/hmg/ddu378 25055869 PMC4240206

[B62] La Spada A. R. Taylor J. P. (2010). Repeat expansion disease: Progress and puzzles in disease pathogenesis. Nat. Rev. Genet. 11 (4), 247–258. PubMed PMID: 20177426; PubMed Central PMCID: PMC4704680. 10.1038/nrg2748 20177426 PMC4704680

[B63] LaCroix A. J. Stabley D. Sahraoui R. Adam M. P. Mehaffey M. Kernan K. (2019). GGC repeat expansion and exon 1 methylation of XYLT1 is a common pathogenic variant in baratela-scott syndrome. Am. J. Hum. Genet. 104 (1), 35–44. Epub 20181213PubMed PMID: 30554721; PubMed Central PMCID: PMC6323552. 10.1016/j.ajhg.2018.11.005 30554721 PMC6323552

[B64] Li Y. Stockton M. E. Eisinger B. E. Zhao Y. Miller J. L. Bhuiyan I. (2018). Reducing histone acetylation rescues cognitive deficits in a mouse model of Fragile X syndrome. Nat. Commun. 9 (1), 2494. Epub 20180627PubMed PMID: 29950602; PubMed Central PMCID: PMC6021376. 10.1038/s41467-018-04869-3 29950602 PMC6021376

[B65] Liu X. S. Wu H. Krzisch M. Wu X. Graef J. Muffat J. (2018). Rescue of fragile X syndrome neurons by DNA methylation editing of the FMR1 gene. Cell 172 (5), 979–992. e6. Epub 20180215PubMed PMID: 29456084; PubMed Central PMCID: PMC6375087. 10.1016/j.cell.2018.01.012 29456084 PMC6375087

[B66] Liufu T. Zheng Y. Yu J. Yuan Y. Wang Z. Deng J. (2022). The polyG diseases: A new disease entity. Acta Neuropathol. Commun. 10 (1), 79. Epub 20220531PubMed PMID: 35642014. 10.1186/s40478-022-01383-y 35642014 PMC9153130

[B67] Lokanga R. A. Entezam A. Kumari D. Yudkin D. Qin M. Smith C. B. (2013). Somatic expansion in mouse and human carriers of fragile X premutation alleles. Hum. Mutat. 34 (1), 157–166. Epub 20121004PubMed PMID: 22887750; PubMed Central PMCID: PMC3524353. 10.1002/humu.22177 22887750 PMC3524353

[B68] Lokanga R. A. Senejani A. G. Sweasy J. B. Usdin K. (2015). Heterozygosity for a hypomorphic Polβ mutation reduces the expansion frequency in a mouse model of the Fragile X-related disorders. PLoS Genet. 11 (4), e1005181. Epub 20150417PubMed PMID: 25886163; PubMed Central PMCID: PMC4401650. 10.1371/journal.pgen.1005181 25886163 PMC4401650

[B69] Lokanga R. A. Zhao X. N. Usdin K. (2014). The mismatch repair protein MSH2 is rate limiting for repeat expansion in a fragile X premutation mouse model. Hum. Mutat. 35 (1), 129–136. PubMed PMID: 24130133; PubMed Central PMCID: PMC3951054. 10.1002/humu.22464 24130133 PMC3951054

[B70] Loomis E. W. Sanz L. A. Chédin F. Hagerman P. J. (2014). Transcription-associated R-loop formation across the human FMR1 CGG-repeat region. PLoS Genet. 10 (4), e1004294. Epub 20140417PubMed PMID: 24743386; PubMed Central PMCID: PMC3990486. 10.1371/journal.pgen.1004294 24743386 PMC3990486

[B71] López Castel A. Cleary J. D. Pearson C. E. (2010). Repeat instability as the basis for human diseases and as a potential target for therapy. Nat. Rev. Mol. Cell Biol. 11 (3), 165–170. PubMed PMID: 20177394. 10.1038/nrm2854 20177394

[B72] Ma D. Tan Y. J. Ng A. S. L. Ong H. L. Sim W. Lim W. K. (2020). Association of NOTCH2NLC repeat expansions with Parkinson disease. JAMA Neurol. 77 (12), 1559–1563. PubMed PMID: 32852534; PubMed Central PMCID: PMC7445625. 10.1001/jamaneurol.2020.3023 32852534 PMC7445625

[B73] Malgowska M. Gudanis D. Kierzek R. Wyszko E. Gabelica V. Gdaniec Z. (2014). Distinctive structural motifs of RNA G-quadruplexes composed of AGG, CGG and UGG trinucleotide repeats. Nucleic Acids Res. 42 (15), 10196–10207. Epub 20140731PubMed PMID: 25081212; PubMed Central PMCID: PMC4150804. 10.1093/nar/gku710 25081212 PMC4150804

[B74] Mila M. Alvarez-Mora M. I. Madrigal I. Rodriguez-Revenga L. (2018). Fragile X syndrome: An overview and update of the FMR1 gene. Clin. Genet. 93 (2), 197–205. Epub 2017/06/16PubMed PMID: 28617938. 10.1111/cge.13075 28617938

[B75] Miller C. J. Kim G. Y. Zhao X. Usdin K. (2020). All three mammalian MutL complexes are required for repeat expansion in a mouse cell model of the Fragile X-related disorders. PLoS Genet. 16 (6), e1008902. Epub 20200626PubMed PMID: 32589669; PubMed Central PMCID: PMC7347238. 10.1371/journal.pgen.1008902 32589669 PMC7347238

[B76] Mitas M. Yu A. Dill J. Haworth I. S. (1995). The trinucleotide repeat sequence d(CGG)15 forms a heat-stable hairpin containing Gsyn. Ganti base pairs. Biochemistry 34 (39), 12803–12811. PubMed PMID: 7548035. 10.1021/bi00039a041 7548035

[B77] Mor-Shaked H. Eiges R. (2018). Reevaluation of FMR1 hypermethylation timing in fragile X syndrome. Front. Mol. Neurosci. 11, 31. Epub 20180206PubMed PMID: 29467618; PubMed Central PMCID: PMC5808132. 10.3389/fnmol.2018.00031 29467618 PMC5808132

[B78] Mori F. Tanji K. Odagiri S. Toyoshima Y. Yoshida M. Ikeda T. (2012). Ubiquilin immunoreactivity in cytoplasmic and nuclear inclusions in synucleinopathies, polyglutamine diseases and intranuclear inclusion body disease. Acta Neuropathol. 124 (1), 149–151. Epub 20120603PubMed PMID: 22661321. 10.1007/s00401-012-0999-z 22661321

[B79] Murat P. Guilbaud G. Sale J. E. (2020). DNA polymerase stalling at structured DNA constrains the expansion of short tandem repeats. Genome Biol. 21 (1), 209. Epub 20200821PubMed PMID: 32819438; PubMed Central PMCID: PMC7441554. 10.1186/s13059-020-02124-x 32819438 PMC7441554

[B80] Nadel Y. Weisman-Shomer P. Fry M. (1995). The fragile X syndrome single strand d(CGG)n nucleotide repeats readily fold back to form unimolecular hairpin structures. J. Biol. Chem. 270 (48), 28970–28977. PubMed PMID: 7499428. 10.1074/jbc.270.48.28970 7499428

[B81] Naumann A. Hochstein N. Weber S. Fanning E. Doerfler W. (2009). A distinct DNA-methylation boundary in the 5'- upstream sequence of the FMR1 promoter binds nuclear proteins and is lost in fragile X syndrome. Am. J. Hum. Genet. 85 (5), 606–616. Epub 20091022PubMed PMID: 19853235; PubMed Central PMCID: PMC2775827. 10.1016/j.ajhg.2009.09.018 19853235 PMC2775827

[B82] Naumann A. Kraus C. Hoogeveen A. Ramirez C. M. Doerfler W. (2014). Stable DNA methylation boundaries and expanded trinucleotide repeats: Role of DNA insertions. J. Mol. Biol. 426 (14), 2554–2566. Epub 20140506PubMed PMID: 24816393. 10.1016/j.jmb.2014.04.025 24816393

[B83] Nolin S. L. Glicksman A. Tortora N. Allen E. Macpherson J. Mila M. (2019). Expansions and contractions of the FMR1 CGG repeat in 5, 508 transmissions of normal, intermediate, and premutation alleles. Am. J. Med. Genet. A 179 (7), 1148–1156. Epub 20190502PubMed PMID: 31050164; PubMed Central PMCID: PMC6619443. 10.1002/ajmg.a.61165 31050164 PMC6619443

[B84] Nolin S. L. Sah S. Glicksman A. Sherman S. L. Allen E. Berry-Kravis E. (2013). Fragile X AGG analysis provides new risk predictions for 45-69 repeat alleles. Am. J. Med. Genet. A 161A (4), 771–778. Epub 20130226PubMed PMID: 23444167; PubMed Central PMCID: PMC4396070. 10.1002/ajmg.a.35833 23444167 PMC4396070

[B85] Oberlé I. Rousseau F. Heitz D. Kretz C. Devys D. Hanauer A. (1991). Instability of a 550-base pair DNA segment and abnormal methylation in fragile X syndrome. Science 252 (5009), 1097–1102. PubMed PMID: 2031184. 10.1126/science.252.5009.1097 2031184

[B86] Okubo M. Doi H. Fukai R. Fujita A. Mitsuhashi S. Hashiguchi S. (2019). GGC repeat expansion of NOTCH2NLC in adult patients with leukoencephalopathy. Ann. Neurol. 86 (6), 962–968. Epub 20191022PubMed PMID: 31433517. 10.1002/ana.25586 31433517

[B87] Pluciennik A. Burdett V. Baitinger C. Iyer R. R. Shi K. Modrich P. (2013). Extrahelical (CAG)/(CTG) triplet repeat elements support proliferating cell nuclear antigen loading and MutLα endonuclease activation. Proc. Natl. Acad. Sci. U. S. A. 110 (30), 12277–12282. Epub 20130709PubMed PMID: 23840062; PubMed Central PMCID: PMC3725108. 10.1073/pnas.1311325110 23840062 PMC3725108

[B88] Poggi L. Richard G. F. (2021). Alternative DNA structures *in vivo*: Molecular evidence and remaining questions. Microbiol. Mol. Biol. Rev. 85 (1), e00110-20. Epub 20201223PubMed PMID: 33361270; PubMed Central PMCID: PMC8549851. 10.1128/MMBR.00110-20 33361270 PMC8549851

[B89] Powell W. T. Coulson R. L. Gonzales M. L. Crary F. K. Wong S. S. Adams S. (2013). R-loop formation at Snord116 mediates topotecan inhibition of Ube3a-antisense and allele-specific chromatin decondensation. Proc. Natl. Acad. Sci. U. S. A. 110 (34), 13938–13943. Epub 20130805PubMed PMID: 23918391; PubMed Central PMCID: PMC3752217. 10.1073/pnas.1305426110 23918391 PMC3752217

[B90] Qurashi A. Li W. Zhou J. Y. Peng J. Jin P. (2011). Nuclear accumulation of stress response mRNAs contributes to the neurodegeneration caused by Fragile X premutation rCGG repeats. PLoS Genet. 7 (6), e1002102. Epub 2011/06/02PubMed PMID: 21655086; PubMed Central PMCID: PMC3107199. 10.1371/journal.pgen.1002102 21655086 PMC3107199

[B91] Qurashi A. Liu H. Ray L. Nelson D. L. Duan R. Jin P. (2012). Chemical screen reveals small molecules suppressing fragile X premutation rCGG repeat-mediated neurodegeneration in Drosophila. Hum. Mol. Genet. 21 (9), 2068–2075. Epub 2012/02/01PubMed PMID: 22298836; PubMed Central PMCID: PMC3315210. 10.1093/hmg/dds024 22298836 PMC3315210

[B92] Reddy K. Schmidt M. H. Geist J. M. Thakkar N. P. Panigrahi G. B. Wang Y. H. (2014). Processing of double-R-loops in (CAG)·(CTG) and C9orf72 (GGGGCC)·(GGCCCC) repeats causes instability. Nucleic Acids Res. 42 (16), 10473–10487. Epub 20140821PubMed PMID: 25147206; PubMed Central PMCID: PMC4176329. 10.1093/nar/gku658 25147206 PMC4176329

[B93] Reddy K. Tam M. Bowater R. P. Barber M. Tomlinson M. Nichol Edamura K. (2011). Determinants of R-loop formation at convergent bidirectionally transcribed trinucleotide repeats. Nucleic Acids Res. 39 (5), 1749–1762. Epub 20101104PubMed PMID: 21051337; PubMed Central PMCID: PMC3061079. 10.1093/nar/gkq935 21051337 PMC3061079

[B94] Renčiuk D. Kypr J. Vorlíčková M. (2011). CGG repeats associated with fragile X chromosome form left-handed Z-DNA structure. Biopolymers 95 (3), 174–181. PubMed PMID: 20960567. 10.1002/bip.21555 20960567

[B95] Roberts R. W. Crothers D. M. (1992). Stability and properties of double and triple helices: Dramatic effects of RNA or DNA backbone composition. Science 258 (5087), 1463–1466. PubMed PMID: 1279808. 10.1126/science.1279808 1279808

[B96] Robin G. Lopez J. R. Espinal G. M. Hulsizer S. Hagerman P. J. Pessah I. N. (2017). Calcium dysregulation and Cdk5-ATM pathway involved in a mouse model of fragile X-associated tremor/ataxia syndrome. Hum. Mol. Genet. 26 (14), 2649–2666. Epub 2017/04/27PubMed PMID: 28444183; PubMed Central PMCID: PMC5886271. 10.1093/hmg/ddx148 28444183 PMC5886271

[B97] Salinas-Rios V. Belotserkovskii B. P. Hanawalt P. C. (2011). DNA slip-outs cause RNA polymerase II arrest *in vitro*: Potential implications for genetic instability. Nucleic Acids Res. 39 (17), 7444–7454. Epub 20110611PubMed PMID: 21666257; PubMed Central PMCID: PMC3177194. 10.1093/nar/gkr429 21666257 PMC3177194

[B98] Schmidt M. H. M. Pearson C. E. (2016). Disease-associated repeat instability and mismatch repair. DNA Repair (Amst) 38, 117–126. Epub 20151212PubMed PMID: 26774442. 10.1016/j.dnarep.2015.11.008 26774442

[B99] Schneider A. Winarni T. I. Cabal-Herrera A. M. Bacalman S. Gane L. Hagerman P. (2020). Elevated FMR1-mRNA and lowered FMRP - a double-hit mechanism for psychiatric features in men with FMR1 premutations. Transl. Psychiatry 10 (1), 205. Epub 20200623PubMed PMID: 32576818; PubMed Central PMCID: PMC7311546. 10.1038/s41398-020-00863-w 32576818 PMC7311546

[B100] Sellier C. Buijsen R. A. M. He F. Natla S. Jung L. Tropel P. (2017). Translation of expanded CGG repeats into FMRpolyG is pathogenic and may contribute to fragile X tremor ataxia syndrome. Neuron 93 (2), 331–347. Epub 2017/01/05PubMed PMID: 28065649; PubMed Central PMCID: PMC5263258. 10.1016/j.neuron.2016.12.016 28065649 PMC5263258

[B101] Sellier C. Freyermuth F. Tabet R. Tran T. He F. Ruffenach F. (2013). Sequestration of DROSHA and DGCR8 by expanded CGG RNA repeats alters microRNA processing in fragile X-associated tremor/ataxia syndrome. Cell Rep. 3 (3), 869–880. Epub 2013/03/07PubMed PMID: 23478018; PubMed Central PMCID: PMC3639429. 10.1016/j.celrep.2013.02.004 23478018 PMC3639429

[B102] Sellier C. Rau F. Liu Y. Tassone F. Hukema R. K. Gattoni R. (2010). Sam68 sequestration and partial loss of function are associated with splicing alterations in FXTAS patients. EMBO J. 29 (7), 1248–1261. Epub 2010/02/25PubMed PMID: 20186122; PubMed Central PMCID: PMC2857464. 10.1038/emboj.2010.21 20186122 PMC2857464

[B103] Sherman S. L. Curnow E. C. Easley C. A. Jin P. Hukema R. K. Tejada M. I. (2014). Use of model systems to understand the etiology of fragile X-associated primary ovarian insufficiency (FXPOI). J. Neurodev. Disord. 6 (1), 26. Epub 20140813PubMed PMID: 25147583; PubMed Central PMCID: PMC4139715. 10.1186/1866-1955-6-26 25147583 PMC4139715

[B104] Skourti-Stathaki K. Torlai Triglia E. Warburton M. Voigt P. Bird A. Pombo A. (2019). R-loops enhance polycomb repression at a subset of developmental regulator genes. Mol. Cell 73 (5), 930–945. e4. Epub 20190129PubMed PMID: 30709709; PubMed Central PMCID: PMC6414425. 10.1016/j.molcel.2018.12.016 30709709 PMC6414425

[B105] Sobczak K. de Mezer M. Michlewski G. Krol J. Krzyzosiak W. J. (2003). RNA structure of trinucleotide repeats associated with human neurological diseases. Nucleic Acids Res. 31 (19), 5469–5482. PubMed PMID: 14500809; PubMed Central PMCID: PMC206466. 10.1093/nar/gkg766 14500809 PMC206466

[B106] Sofola O. A. Jin P. Botas J. Nelson D. L. (2007). Argonaute-2-dependent rescue of a Drosophila model of FXTAS by FRAXE premutation repeat. Hum. Mol. Genet. 16 (19), 2326–2332. Epub 2007/07/17PubMed PMID: 17635840. 10.1093/hmg/ddm186 17635840

[B107] Sone J. Mitsuhashi S. Fujita A. Mizuguchi T. Hamanaka K. Mori K. (2019). Long-read sequencing identifies GGC repeat expansions in NOTCH2NLC associated with neuronal intranuclear inclusion disease. Nat. Genet. 51 (8), 1215–1221. Epub 20190722PubMed PMID: 31332381. 10.1038/s41588-019-0459-y 31332381

[B108] Su X. A. Freudenreich C. H. (2017). Cytosine deamination and base excision repair cause R-loop-induced CAG repeat fragility and instability in *Saccharomyces cerevisiae*. Proc. Natl. Acad. Sci. U. S. A. 114 (40), E8392–E401. Epub 20170918PubMed PMID: 28923949; PubMed Central PMCID: PMC5635916. 10.1073/pnas.1711283114 28923949 PMC5635916

[B109] Sullivan S. D. Welt C. Sherman S. (2011). FMR1 and the continuum of primary ovarian insufficiency. Semin. Reprod. Med. 29 (4), 299–307. Epub 20111003PubMed PMID: 21969264. 10.1055/s-0031-1280915 21969264

[B110] Sun Q. Y. Xu Q. Tian Y. Hu Z. M. Qin L. X. Yang J. X. (2020). Expansion of GGC repeat in the human-specific NOTCH2NLC gene is associated with essential tremor. Brain 143 (1), 222–233. PubMed PMID: 31819945. 10.1093/brain/awz372 31819945

[B111] Tabet R. Schaeffer L. Freyermuth F. Jambeau M. Workman M. Lee C. Z. (2018). CUG initiation and frameshifting enable production of dipeptide repeat proteins from ALS/FTD C9ORF72 transcripts. Nat. Commun. 9 (1), 152. Epub 20180111PubMed PMID: 29323119; PubMed Central PMCID: PMC5764992. 10.1038/s41467-017-02643-5 29323119 PMC5764992

[B112] Takahashi S. Sugimoto N. (2020). Stability prediction of canonical and non-canonical structures of nucleic acids in various molecular environments and cells. Chem. Soc. Rev. 49 (23), 8439–8468. Epub 20201013PubMed PMID: 33047751. 10.1039/d0cs00594k 33047751

[B113] Tassone F. Beilina A. Carosi C. Albertosi S. Bagni C. Li L. (2007). Elevated FMR1 mRNA in premutation carriers is due to increased transcription. RNA 13 (4), 555–562. Epub 20070205PubMed PMID: 17283214; PubMed Central PMCID: PMC1831862. 10.1261/rna.280807 17283214 PMC1831862

[B114] Tassone F. Hagerman R. J. Taylor A. K. Gane L. W. Godfrey T. E. Hagerman P. J. (2000). Elevated levels of FMR1 mRNA in carrier males: A new mechanism of involvement in the fragile-X syndrome. Am. J. Hum. Genet. 66 (1), 6–15. PubMed PMID: 10631132; PubMed Central PMCID: PMC1288349. 10.1086/302720 10631132 PMC1288349

[B115] Tassone F. Iwahashi C. Hagerman P. J. (2004). FMR1 RNA within the intranuclear inclusions of fragile X-associated tremor/ataxia syndrome (FXTAS). RNA Biol. 1 (2), 103–105. Epub 2004/07/17PubMed PMID: 17179750. 10.4161/rna.1.2.1035 17179750

[B116] Tian Y. Wang J. L. Huang W. Zeng S. Jiao B. Liu Z. (2019). Expansion of human-specific GGC repeat in neuronal intranuclear inclusion disease-related disorders. Am. J. Hum. Genet. 105 (1), 166–176. Epub 20190606PubMed PMID: 31178126; PubMed Central PMCID: PMC6612530. 10.1016/j.ajhg.2019.05.013 31178126 PMC6612530

[B117] Todd P. K. Oh S. Y. Krans A. He F. Sellier C. Frazer M. (2013). CGG repeat-associated translation mediates neurodegeneration in fragile X tremor ataxia syndrome. Neuron 78 (3), 440–455. Epub 2013/04/18PubMed PMID: 23602499; PubMed Central PMCID: PMC3831531. 10.1016/j.neuron.2013.03.026 23602499 PMC3831531

[B118] Todd P. K. Oh S. Y. Krans A. Pandey U. B. Di Prospero N. A. Min K. T. (2010). Histone deacetylases suppress CGG repeat-induced neurodegeneration via transcriptional silencing in models of fragile X tremor ataxia syndrome. PLoS Genet. 6 (12), e1001240. Epub 2010/12/09PubMed PMID: 21170301; PubMed Central PMCID: PMC3000359. 10.1371/journal.pgen.1001240 21170301 PMC3000359

[B119] Todd P. K. Paulson H. L. (2010). RNA-mediated neurodegeneration in repeat expansion disorders. Ann. Neurol. 67 (3), 291–300. PubMed PMID: 20373340; PubMed Central PMCID: PMC2852186. 10.1002/ana.21948 20373340 PMC2852186

[B120] Tran T. Childs-Disney J. L. Liu B. Guan L. Rzuczek S. Disney M. D. (2014). Targeting the r(CGG) repeats that cause FXTAS with modularly assembled small molecules and oligonucleotides. ACS Chem. Biol. 9 (4), 904–912. Epub 2014/02/11PubMed PMID: 24506227; PubMed Central PMCID: PMC4287843. 10.1021/cb400875u 24506227 PMC4287843

[B121] Usdin K. Woodford K. J. (1995). CGG repeats associated with DNA instability and chromosome fragility form structures that block DNA synthesis *in vitro* . Nucleic Acids Res. 23 (20), 4202–4209. PubMed PMID: 7479085; PubMed Central PMCID: PMC307363. 10.1093/nar/23.20.4202 7479085 PMC307363

[B122] Verkerk A. J. Pieretti M. Sutcliffe J. S. Fu Y. H. Kuhl D. P. Pizzuti A. (1991). Identification of a gene (FMR-1) containing a CGG repeat coincident with a breakpoint cluster region exhibiting length variation in fragile X syndrome. Cell 65 (5), 905–914. PubMed PMID: 1710175. 10.1016/0092-8674(91)90397-h 1710175

[B123] Verma A. K. Khan E. Mishra S. K. Jain N. Kumar A. (2019). Piperine modulates protein mediated toxicity in fragile X-associated tremor/ataxia syndrome through interacting expanded CGG repeat (r(CGG)exp) RNA. ACS Chem. Neurosci. 10 (8), 3778–3788. Epub 2019/07/02PubMed PMID: 31264835. 10.1021/acschemneuro.9b00282 31264835

[B124] Verma A. K. Khan E. Mishra S. K. Mishra A. Charlet-Berguerand N. Kumar A. (2020). Curcumin regulates the r(CGG)exp RNA hairpin structure and ameliorate defects in fragile X-associated tremor ataxia syndrome. Front. Neurosci. 14, 295. Epub 2020/04/07PubMed PMID: 32317919; PubMed Central PMCID: PMC7155420. 10.3389/fnins.2020.00295 32317919 PMC7155420

[B125] Viguera E. Canceill D. Ehrlich S. D. (2001). Replication slippage involves DNA polymerase pausing and dissociation. EMBO J. 20 (10), 2587–2595. PubMed PMID: 11350948; PubMed Central PMCID: PMC125466. 10.1093/emboj/20.10.2587 11350948 PMC125466

[B126] Wang Y. H. (2007). Chromatin structure of repeating CTG/CAG and CGG/CCG sequences in human disease. Front. Biosci. 12, 4731–4741. Epub 20070501PubMed PMID: 17485409. 10.2741/2422 17485409

[B127] Wang Y. H. Gellibolian R. Shimizu M. Wells R. D. Griffith J. (1996). Long CCG triplet repeat blocks exclude nucleosomes: A possible mechanism for the nature of fragile sites in chromosomes. J. Mol. Biol. 263 (4), 511–516. PubMed PMID: 8918933. 10.1006/jmbi.1996.0593 8918933

[B128] Weisman-Shomer P. Cohen E. Fry M. (2002). Distinct domains in the CArG-box binding factor A destabilize tetraplex forms of the fragile X expanded sequence d(CGG)n. Nucleic Acids Res. 30 (17), 3672–3681. PubMed PMID: 12202751; PubMed Central PMCID: PMC137428. 10.1093/nar/gkf506 12202751 PMC137428

[B129] Weisman-Shomer P. Cohen E. Hershco I. Khateb S. Wolfovitz-Barchad O. Hurley L. H. (2003). The cationic porphyrin TMPyP4 destabilizes the tetraplex form of the fragile X syndrome expanded sequence d(CGG)n. Nucleic Acids Res. 31 (14), 3963–3970. PubMed PMID: 12853612; PubMed Central PMCID: PMC165968. 10.1093/nar/gkg453 12853612 PMC165968

[B130] Willemsen R. Bontekoe C. J. Severijnen L. A. Oostra B. A. (2002). Timing of the absence of FMR1 expression in full mutation chorionic villi. Hum. Genet. 110 (6), 601–605. Epub 20020416PubMed PMID: 12107447. 10.1007/s00439-002-0723-5 12107447

[B131] Winnepenninckx B. Debacker K. Ramsay J. Smeets D. Smits A. FitzPatrick D. R. (2007). CGG-repeat expansion in the DIP2B gene is associated with the fragile site FRA12A on chromosome 12q13.1. Am. J. Hum. Genet. 80 (2), 221–231. Epub 20061212PubMed PMID: 17236128; PubMed Central PMCID: PMC1785358. 10.1086/510800 17236128 PMC1785358

[B132] Xu K. Li Y. Allen E. G. Jin P. (2021). Therapeutic development for CGG repeat expansion-associated neurodegeneration. Front. Cell. Neurosci. 15, 655568. Epub 20210512PubMed PMID: 34054431; PubMed Central PMCID: PMC8149615. 10.3389/fncel.2021.655568 34054431 PMC8149615

[B133] Yang B. Rodgers M. T. (2014). Base-pairing energies of proton-bound heterodimers of cytosine and modified cytosines: Implications for the stability of DNA i-motif conformations. J. Am. Chem. Soc. 136 (1), 282–290. Epub 20131219PubMed PMID: 24320604. 10.1021/ja409515v 24320604

[B134] Yrigollen C. M. Martorell L. Durbin-Johnson B. Naudo M. Genoves J. Murgia A. (2014). AGG interruptions and maternal age affect FMR1 CGG repeat allele stability during transmission. J. Neurodev. Disord. 6 (1), 24. Epub 20140730PubMed PMID: 25110527; PubMed Central PMCID: PMC4126815. 10.1186/1866-1955-6-24 25110527 PMC4126815

[B135] Yu A. Barron M. D. Romero R. M. Christy M. Gold B. Dai J. (1997). At physiological pH, d(CCG)15 forms a hairpin containing protonated cytosines and a distorted helix. Biochemistry 36 (12), 3687–3699. PubMed PMID: 9132022. 10.1021/bi9625410 9132022

[B136] Yu J. Deng J. Guo X. Shan J. Luan X. Cao L. (2021). The GGC repeat expansion in NOTCH2NLC is associated with oculopharyngodistal myopathy type 3. Brain. 144 (6), 1819–1832. PubMed PMID: 33693509; PubMed Central PMCID: PMC8320266. 10.1093/brain/awab077 33693509 PMC8320266

[B137] Yu J. Shan J. Yu M. Di L. Xie Z. Zhang W. (2022). The CGG repeat expansion in RILPL1 is associated with oculopharyngodistal myopathy type 4. Am. J. Hum. Genet. 109 (3), 533–541. Epub 20220210PubMed PMID: 35148830; PubMed Central PMCID: PMC8948162. 10.1016/j.ajhg.2022.01.012 35148830 PMC8948162

[B138] Zhao X. Usdin K. (2021). (Dys)function follows form: Nucleic acid structure, repeat expansion, and disease pathology in *FMR1* disorders. Int. J. Mol. Sci. 22 (17), 9167. Epub 20210825PubMed PMID: 34502075; PubMed Central PMCID: PMC8431139. 10.3390/ijms22179167 34502075 PMC8431139

[B139] Zhao X. Zhang Y. Wilkins K. Edelmann W. Usdin K. (2018). MutLγ promotes repeat expansion in a Fragile X mouse model while EXO1 is protective. PLoS Genet. 14 (10), e1007719. Epub 20181012PubMed PMID: 30312299; PubMed Central PMCID: PMC6200270. 10.1371/journal.pgen.1007719 30312299 PMC6200270

[B140] Zhao X. N. Kumari D. Gupta S. Wu D. Evanitsky M. Yang W. (2015). Mutsβ generates both expansions and contractions in a mouse model of the Fragile X-associated disorders. Hum. Mol. Genet. 24 (24), 7087–7096. Epub 20150929PubMed PMID: 26420841; PubMed Central PMCID: PMC4654059. 10.1093/hmg/ddv408 26420841 PMC4654059

[B141] Zhao X. N. Lokanga R. Allette K. Gazy I. Wu D. Usdin K. (2016). A MutSβ-dependent contribution of MutSα to repeat expansions in fragile X premutation mice? PLoS Genet. 12 (7), e1006190. Epub 20160718PubMed PMID: 27427765; PubMed Central PMCID: PMC4948851. 10.1371/journal.pgen.1006190 27427765 PMC4948851

[B142] Zhu Q. Jiang J. Gendron T. F. McAlonis-Downes M. Jiang L. Taylor A. (2020). Reduced C9ORF72 function exacerbates gain of toxicity from ALS/FTD-causing repeat expansion in C9orf72. Nat. Neurosci. 23 (5), 615–624. Epub 20200413PubMed PMID: 32284607; PubMed Central PMCID: PMC7384305. 10.1038/s41593-020-0619-5 32284607 PMC7384305

[B143] Zumwalt M. Ludwig A. Hagerman P. J. Dieckmann T. (2007). Secondary structure and dynamics of the r(CGG) repeat in the mRNA of the fragile X mental retardation 1 (FMR1) gene. RNA Biol. 4 (2), 93–100. Epub 20070912PubMed PMID: 17962727. 10.4161/rna.4.2.5039 17962727

